# Genetic evidence linking gastroesophageal reflux disease to chronic kidney disease and kidney failure: a two-step Mendelian randomization study

**DOI:** 10.1080/0886022X.2025.2577842

**Published:** 2025-11-03

**Authors:** Fanghong Zheng, Dan Shen, Ying Lv, Jing Yuan, Yan Zha, Yuqi Yang

**Affiliations:** a Graduate School, Zunyi Medical University, Zunyi, Guizhou, China; b Department of Nephrology, Guizhou Provincial People’s Hospital, Guiyang, China; c NHC Key Laboratory of Pulmonary Immunological Disease, Guizhou Provincial People’s Hospital, Guiyang, China; d Guizhou Provincial Key Laboratory of Pathogenesis and Prevention of Common Chronic Diseases Research, Guizhou Provincial People’s Hospital, Guiyang, China

**Keywords:** Chronic kidney disease progression, kidney failure, mendelian randomization, mediation, diabetes, blood pressure

## Abstract

To investigate the causal association between gastroesophageal reflux disease (GERD) and chronic kidney disease (CKD) progression and its potential metabolic mediators. Summary-level data were extracted from the overall genome-wide association studies of the FinnGen and UK Biobank databases. This study employed two-sample, bidirectional, two-step, and multivariable Mendelian randomization (MR) techniques, utilizing single-nucleotide polymorphisms (SNPs) as genetic instruments for exposure and mediators, thereby minimizing bias due to confounders and reverse causation. We harnessed summary-level data from a genome-wide association study of GERD, proposed mediators, and CKD progression, including CKD, kidney failure, and dialysis-dependent kidney failure. The total effect of GERD on CKD progression was decomposed into direct effects and indirect effects through multiple mediators. GERD was associated with an increased risk of CKD (odds ratio [OR]: 1.18, 95% confidence interval [CI]: 1.05–1.33), kidney failure (1.23, 1.11–1.36), dialysis-dependent kidney failure (1.26, 1.19–1.34), whereas the reverse causality hypothesis did not hold. Type 2 diabetes mellitus (T2DM) mediated 14.33%–43.24% of the effect of GERD on CKD progression, which was followed by systolic blood pressure (mediation: 3.85%–5.46%). These results supported a potentially causal damage effect of GERD against CKD progression, which T2DM and SBP considerably mediate. Interventions with these factors could significantly decrease the burden of CKD attributable to GERD.

## Graphical abstract

**Figure:**
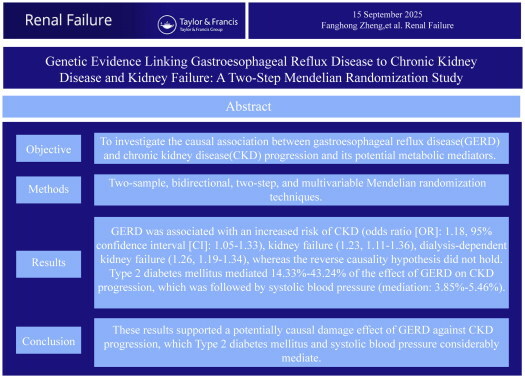

## Introduction

Chronic Kidney Disease (CKD) has emerged as an increasingly prominent global health issue that is characterized by a high prevalence rate, public awareness, and exorbitant medical costs, and it is one of the leading causes of death worldwide [1–3]. It significantly contributed to cardiovascular comorbidities, cachexia, anemia, and impaired digestive system [4,5]. Except for traditional risk factors, including obesity, diabetes, hypertension, cardiovascular diseases, hyperlipidemia, and high body mass index (BMI), gastroesophageal reflux disease (GERD) is also a substantial risk factor for CKD [4,6,7]. GERD is a common digestive disorder worldwide, which affects approximately 20% of the adult population [5]. Previous studies have revealed that the prevalence of GERD in patients with CKD is higher compared to those without CKD (23.5% vs 14.8%), and this difference has been consistently observed across all stages of CKD [3,8]. However, few studies have evaluated the progression of GERD-induced CKD, which is an underappreciated risk factor in CKD. And the underlying mechanism has not been fully revealed. Diabetes, hypertension, hyperlipidemia, and obesity are common risk factors for both, which may promote CKD progression [6,9,10]. Thus, it is critical to recognize modifiable risk factors mediating the association of GERD with CKD progression.

Previous data came from clinical observational studies, which could not prove a causal effect. Mendelian randomization (MR) is an application of instrumental variable analysis, which aims to test a causal hypothesis in non-experimental data [11]. This approach uses the genetic variants associated with lifelong exposure of interest to assess the magnitude of the effect of genetically predicted exposures on outcomes [7]. Previous Mendelian studies have found causal relationships between GERD and lung cancer as well as rheumatoid arthritis [12,13]. However, no research has evaluated the causal relationship between GERD and CKD progression.

In our study, we used MR to reveal the potential causal effect of GERD on CKD progression and to evaluate the mediating pathways underlying the association between the two diseases. For the mediating factors, we investigated the mediating roles of body composition and strength, body fat, blood pressure, glucose metabolism, and lipid and lipoprotein. This will enhance understanding of the interaction between GERD and CKD progression, thereby providing an important guarantee for kidney health, having a cross-system significance to protect the health of organs throughout the body, open up new research dimensions for the development of the discipline, and provide a basis for the formulation of public health intervention strategies and the management of high-risk groups.

## Materials and methods

### Study design

**Figure 1.**
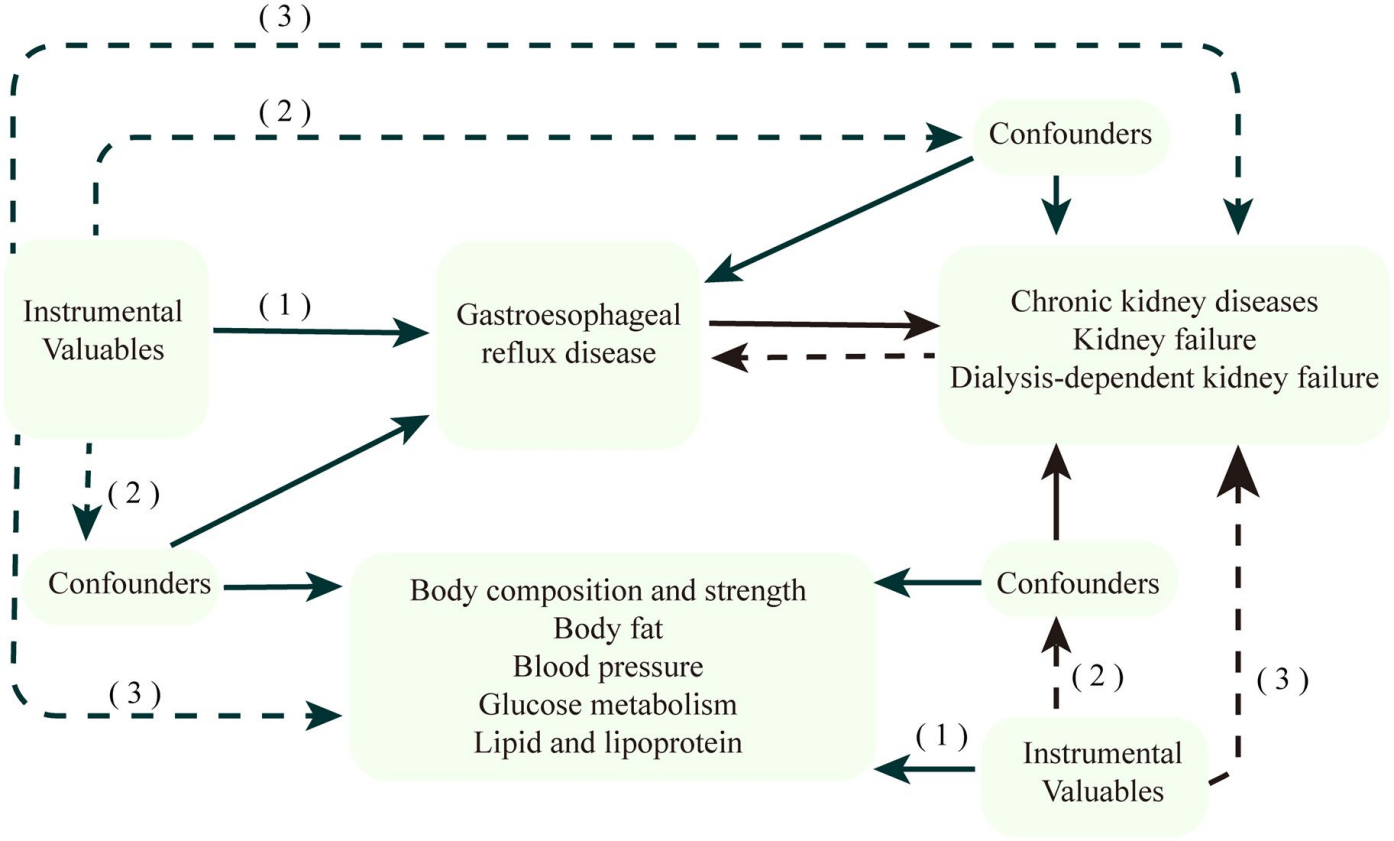
The flow chart of the study is based on three assumptions: (1) the IVs must be significantly connected to the exposure; (2) the IVs cannot be connected to any known confounders that could alter the association between an exposure and an outcome; and (3) the IVs must be unrelated to the outcomes and may only affect the outcomes through their effects on the exposure. This Figure illustrates a diagram of our MR study. The dashed lines indicate irrelevance, and the solid lines indicate relevance.

Figure 1 displayed the diagram of the study design. The results of this research revealed the potential causal relationships between GERD and the risk of CKD progression, which we illustrated using a two-sample MR approach. Reverse MR Analysis eliminates the opportunity of reverse causality of CKD progression, causing GERD. In addition, this research has used a multivariable MR technique to recognize the direct impacts that GERD exerts on CKD progression risk. We have also identified a variety of mediators that influence the link between GERD and CKD progression risk.

MR analysis had three key assumptions. The genetic instruments must satisfy three conditions: (1) The relevance condition accounts for that the instrumental variable (IV) must be substantially associated with the exposure of interest; (2) The assumption of independence mandates that IV must remain unassociated with any confounding factors; (3) The exclusivity assumption suggests that the IV must be independent of the outcome of interest and should influence the outcome purely through the exposure [14]. MR employed single-nucleotide polymorphisms (SNPs) as instrumental variables, which are designed to study the causal effect of an exposure on an outcome.

### Ethical consideration

The analysis used summary data that was released to the public, thus not necessitating approval or informed consent of the institutional review board.

### Data source

This comprehensive data set of GERD included 129,080 cases and 473,524 controls of European descent, which were directly retrieved from the GWAS Catalog (https://www.ebi.ac.uk/gwas/studies/GCST90000514) [14]. SNPs in association with GERD were used as instrumental variables, employing methods including weighted mode (WM), weighted medians, MR-PRESSO, and MR-Egger to solve underlying pleiotropy and confounding factors.

To avoid duplication of samples, we gain the CKD progression datasets from https://www.finngen.fi/en, including a wide array of CKD progression, including CKD (*n* = 363,177), kidney failure (*n* = 363,177), and dialysis-dependent kidney failure (*n* = 363,177). A literature review based on observational studies and MR studies, our candidate mediators included 19 metabolic factors that can be categorized into body composition and strength (BMI, hip circumference, appendicular lean mass, grip strength), body fat (whole body fat mass, body fat percentage, trunk fat mass), blood pressure (hypertension, systolic blood pressure [SBP], diastolic blood pressure [DBP]), glucose metabolism (type 2 diabetes mellitus [T2DM]), and lipid and lipoprotein (total cholesterol, triglycerides, high-density lipoprotein-cholesterol, low-density lipoprotein-cholesterol, docosahexaenoic acid, polyunsaturated fatty acid, monounsaturated fatty acid, omega).

We have identified the changeable mediators of the causal relationship between GERD and CKD progression by the following criteria: (1) GERD should have a causal relationship with the mediator; (2) the mediator should exert a direct causal influence on CKD progression independent of the effect GERD; and (3) the total effect of GERD on CKD progression should be congruent with the mediating role of the mediator. These candidate mediators, being ubiquitously present and amenable to modification, prevention, or treatment, emerge as valuable targets for preventing or intervening in the progression of chronic kidney disease. They also have accessible genetic instruments originating from GWAS. The common databases for the aforementioned mediators’ GWAS were obtained from https://www.nealelab.is/uk-biobank. A comprehensive overview relevant to these data sources is provided in Table 1. The list of elected SNPs used in the research can be found in Additional File 2: Table S1.

**Table 1.** GWAS data source of the MR study.

Phenotype	PMID or GWAS ID	Sample size (overall or case/control)	Ancestry	Unit	Consortium or cohort study
**Exposure**					
GERD	ebi-a-GCST0000514	129080/473524	European	Log-transformed odds	UK Biobank
**Outcome**					
CKD	R9_N14_CHRONKIDNEYDIS	9073/363177	European	Log-transformed odds	FinnGen
Kidney failure	R9_N14_RENEALL	14100/363177	European	Log-transformed odds	FinnGen
Dialysis-dependent kidney failure	dialysis	1004/363177	European	Log-transformed odds	FinnGen
**Candidate mediator**					
Body composition and strength					
BMI	29273807	806,834	European	1-SD	GIANT, UK Biobank
Hip circumference	25673412	225,487	European	1-SD	GIANT
Appendicular lean mass	33097823	450,243	European	1-SD	UK Biobank
Grip strength	34017140	461,089	European	1-SD	MRC-IEU
**Body fat**					
Whole body fat mass	34662886	454,137	European	1-SD	MRC-IEU
Body fat percentage	34662886	454,633	European	1-SD	MRC-IEU
Trunk fat mass	ukb-a-291	331,093	European	1-SD	Neale Lab
**Blood pressure**					
Hypertension	ukb-b-12493	54,358/408,652	European	Log-transformed odds	MRC-IEU
Systolic blood pressure	30224653	757,601	European	mmHg	ICBP
Diastolic blood pressure	30224653	757,601	European	mmHg	ICBP
**Glucose metabolism**					
T2DM	34594039	490,089	European	Log-transformed odds	UK Biobank
**Lipid and lipoprotein**					
Total cholesterol	24097068	188,577	European	1-SD	GLGC
Triglycerides	24097068	188,577	European	1-SD	GLGC
High-density lipoprotein-cholesterol	24097068	188,577	European	1-SD	GLGC
Low-density lipoprotein-cholesterol	24097068	188,577	European	1-SD	GLGC
Docosahexaenoic acid	35213538	115,006	European	1-SD	UK Biobank
Polyunsaturated fatty acid	35213538	115,006	European	1-SD	UK Biobank
Monounsaturated fatty acid	35213538	115,006	European	1-SD	UK Biobank
Omega	35213538	115,006	European	1-SD	UK Biobank

GWAS: genome-wide association study; MR: Mendelian randomization; SD: standard deviation; GERD: gastroesophageal reflux disease; CKD: chronic kidney disease; BMI: body mass index; T2DM: type 2 diabetes mellitus.

In the study, we noted that the dependent and mediator variables were from the UK Biobank database, while the outcome variables were from the Finngen database. Participants in the UK Biobank are predominantly white British and have a relatively uniform genetic background; however, there is still some genetic diversity. In contrast, participants in the Finnish database were predominantly Finnish and had a genetic background that differed significantly from that of white British populations, especially for some specific genetic variants. Such ancestry differences may lead to biased estimates of causal effects. For example, certain genetic variants may have stronger associations with dependent and mediator variables in the British population but may be weaker in the Finnish population, or vice versa. This difference may lead to inaccurate estimates of causal effects. To assess the effect of ancestry differences, we performed sensitivity analyses, which include MR Egger, Weighted median, Weighted mode, and MR-PRESSO.

### Genetic instrument selection

The SNP selection process utilized the above-mentioned GWAS databases and adhered to the key hypothesis of MR. To handle potential problems related to linkage disequilibrium, a stringent clumping procedure was implemented, employing clumping windows of size 10,000 kb and an r^2^ threshold of 0.001. The second minor allele frequency is *>* 0.01. Furthermore, a threshold of *p <* 5 × 10^−8^ was employed to detect SNPs drastically associated with exposure [10]. In addition, an examination for potential confounding factors was conducted on the identified SNPs. SNPs connected with these confounders will be eliminated from this MR study.

To avert potential bias of weak instruments, we employed the *F*-statistic (calculated as *F* = beta^2^/se^2^) to assess the intensity of IV, whereby an *F*-value > 10 was deemed indicative of a sufficiently strong correlation between IVs and exposures to safeguard MR analysis results against weak instrument bias [15]. Data harmonization steps were carried out prior to each MR analysis because the alleles of SNPs influencing both the exposure and the outcome must correspond to the same allele. Furthermore, after excluding SNPs with potential pleiotropy using MR-pleiotropy residual sum and outlier (MR-PRESSO), we performed MR analysis to assess robustness.

### Statistical analysis

Using R programming (version 4.4.2), initially, we employed five distinct methods to probe the potential causal relationship between GERD and CKD progression, including Inverse variance weighted (IVW), MR Egger, Weighted median, Weighted mode, and MR-PRESSO. Among them, the IVW method, which is regarded as the primary analysis approach, was employed [16]. The remaining four methods were employed as secondary analyses. The IVW method allowed us to acquire an unbiased estimate of the current situation without considering horizontal pleiotropy [17], with Cochran’s Q test assessing heterogeneity [18]. We used the weighted median method, which enables us to estimate the underlying bias arising from ineffective instruments [19]. The approach can generate reliable estimates provided that over 50% of the weight in the meta-analysis is based on valid SNPs. The weighted model helps to reduce the influence of outliers or invalid instrumental variables, resulting in a more robust inference of causal effects [20]. MR-Egger was used to determine whether there was horizontal pleiotropy. A p-value of more than 0.05 was regarded as indicating that horizontal pleiotropy was not significant. Compared with MR-Egger, MR-PRESSO showed higher accuracy in detecting horizontal pleiotropy and identifying outliers [6]. SNPs identified as outliers through the MR-PRESSO analysis process will be removed from the study.

A two-step MR analysis was performed to explore the mediation effect of potential mediators on the causal associations between GERD and CKD progression. In the first stage, we applied the univariable MR (UVMR) technique to evaluate the causal influence of GERD on the potential mediator, with each estimate denoted by *β1*. Subsequently, in the second stage, we evaluated the direct effect of potential mediators on CKD progression by the multivariable MR (MVMR) method, controlling for the genetic factor GERD, with each estimate denoted by *β*2. We use *β*0 to denote the total effect estimate of the genetic factor GERD on CKD progression. The mediation proportion was then calculated by dividing the product of *β1* and *β2* by *β0*. To obtain the 95% confidence interval (CI) of the mediating impact, we used the delta method [16]. Reverse causation analysis was also conducted to examine the reverse causal association. The significance level of the statistics was set at 0.05. All MR estimates were presented as odds ratios (ORs) with 95% CIs for binary outcomes and β coefficients with CIs for continuous outcomes. All MR analyses were carried out using R packages Two-Sample MR, MVMR, and MRPRESSO in R software.

### Guarantor

Dr. Yuqi Yang and Fanghong Zheng verified instrument selection, harmonization, pleiotropy diagnostics, and mediation estimation.

## Results

### Causal effects of gastroesophageal reflux disease on chronic kidney disease progression

**Figure 2.**
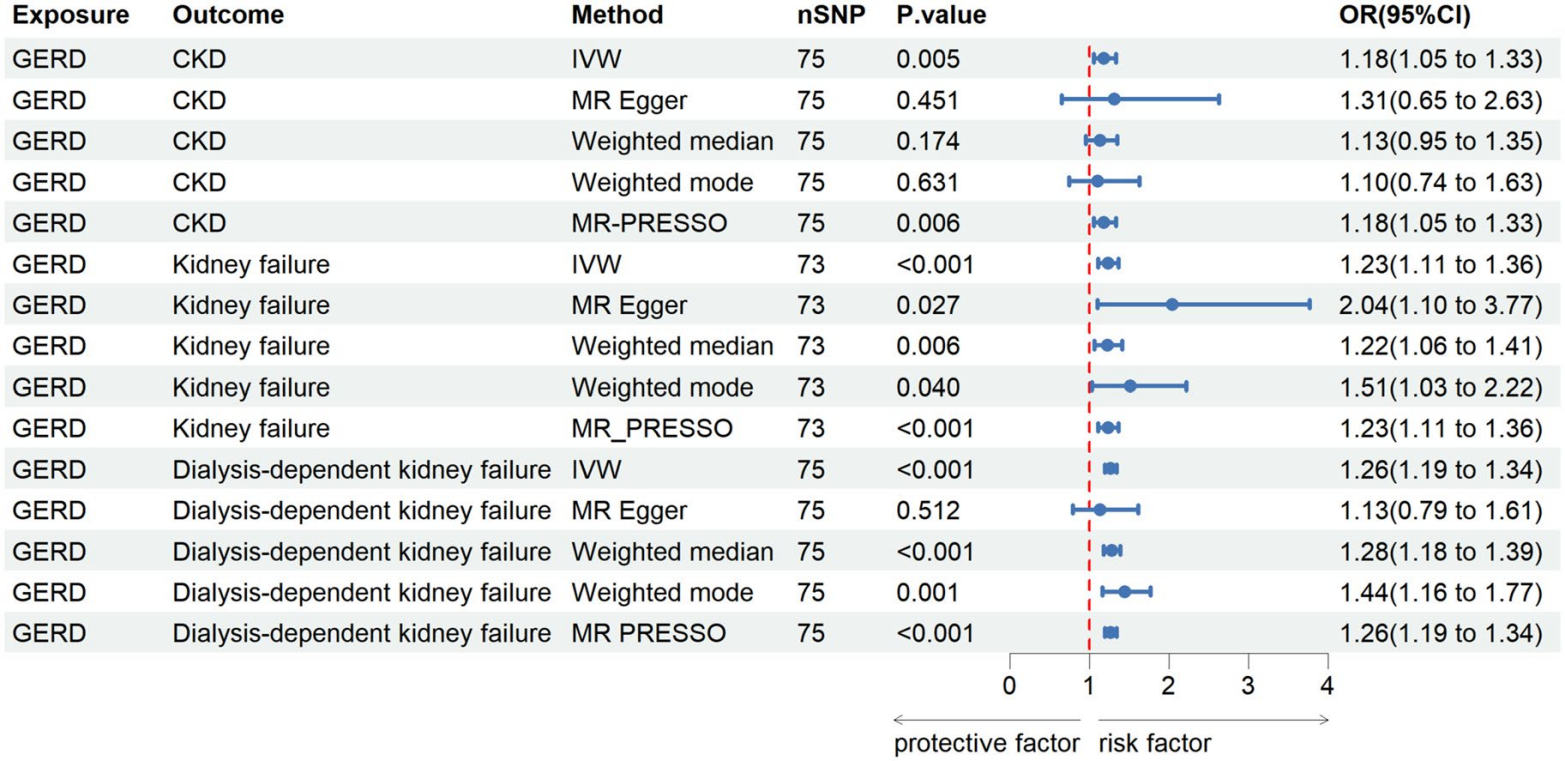
MR analysis of GERD association with CKD progression risk.

The causal effects of GERD on chronic kidney disease progression are shown in Figure 2, enumerated in detail in Additional file 1: Table S1. The primary IVW analysis findings demonstrate a significant association whereby genetic susceptibility to GERD confers an elevated risk of CKD (OR: 1.18, 95%CI: 1.05–1.33, *p* = 0.005), kidney failure (1.23, 1.11–1.36, <0.001), and dialysis-dependent kidney failure(1.26, 1.19–1.34, <0.001).

### Causal effects of chronic kidney disease progression on gastroesophageal reflux disease

**Figure 3.**
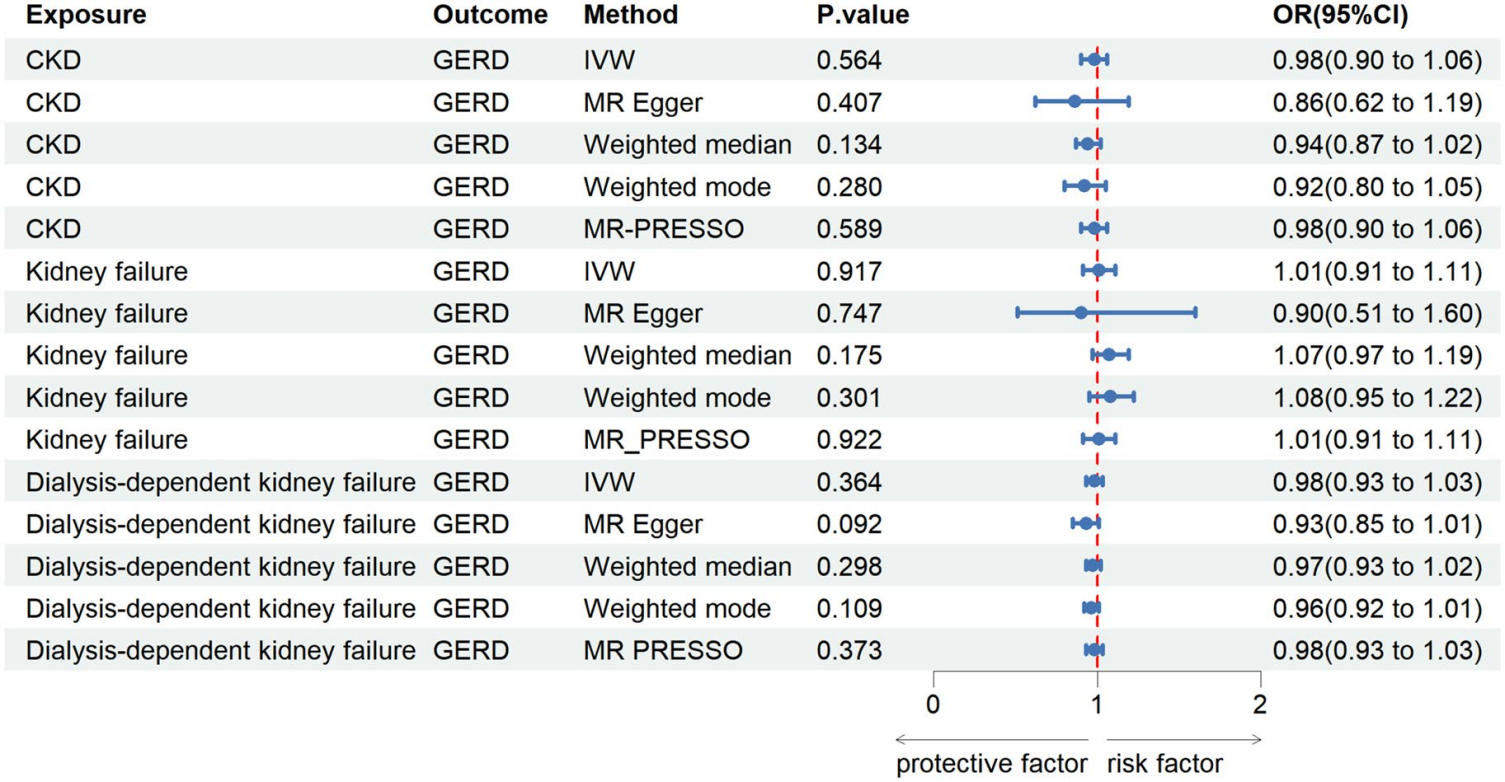
Reverse MR analysis of CKD progression on GERD.

There was no significant association of chronic kidney disease progression with GERD (Figure 3). Several sensitivity analyses still demonstrated no associations (Additional file 1: Table S2).

### Causal effects of gastroesophageal reflux disease on mediators

**Figure 4.**
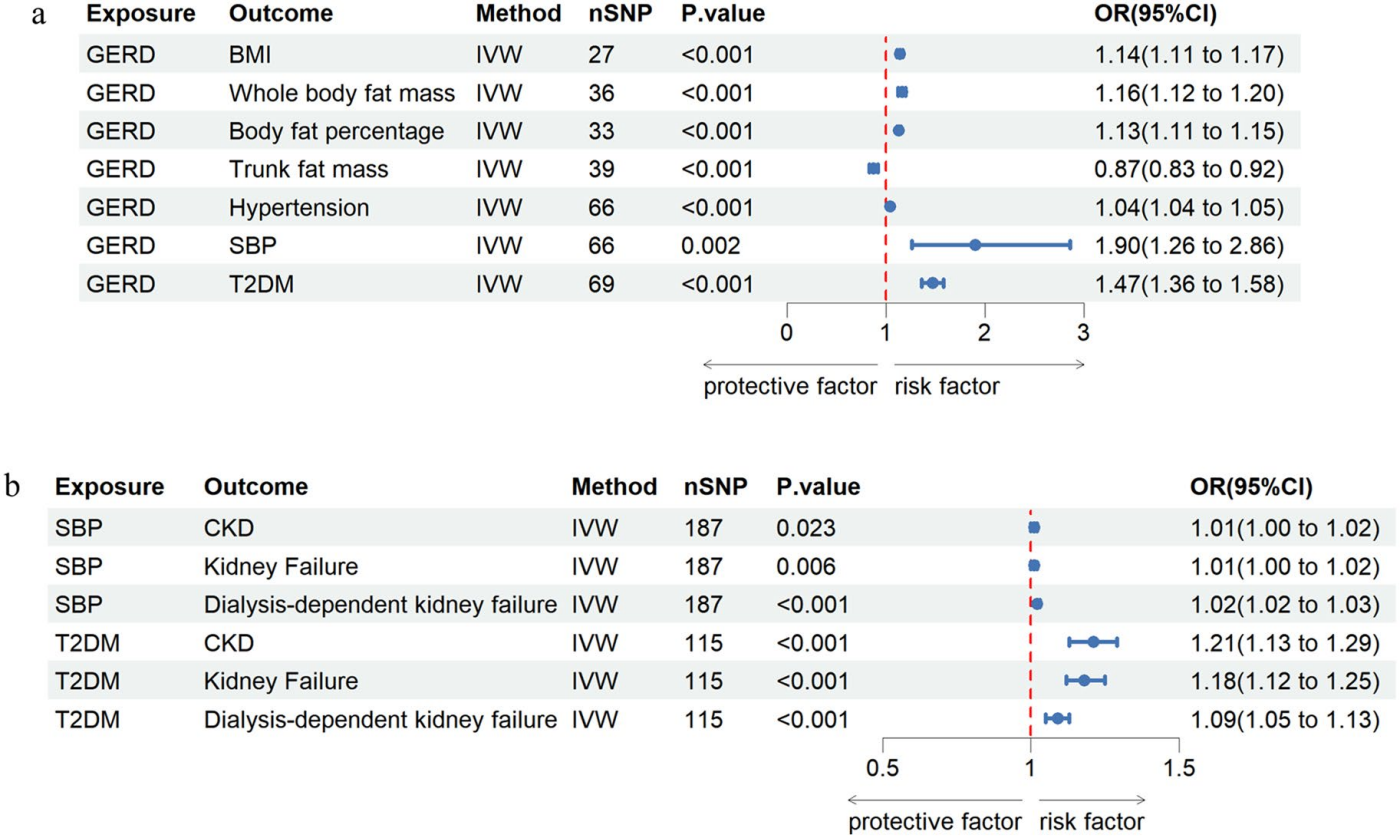
a. The estimates for the causal effect of GERD on mediators. b. The estimates for a causal effect of mediators on CKD progression.

We conducted an MVMR analysis to examine whether the impact of GERD on CKD progression depends on specific covariates. The causal relationship of 19 metabolic factors in GERD was studied by the IVW method. Among the mediating factors affecting GERD, 7 were statistically significant and 12 were not. GERD was positively related to BMI(OR = 1.14, 95% CI: 1.11–1.17), whole body fat mass(OR = 1.16, 95% CI: 1.12–1.20), body fat percentage(OR = 1.13, 95% CI: 1.11–1.15), hypertension (OR = 1.04, 95% CI: 1.04–1.05), systolic blood pressure (OR = 1.90, 95% CI: 1.26–2.86), and type 2 diabetes mellitus (OR = 1.47, 95% CI: 1.36–1.58). In addition, our study results discover a negative correlation between GERD and a factor, which includes trunk fat mass (OR = 0.87, 95% CI: 0.83–0.92). (Figure 4(a), Additional file 1: Table S3).

### Causal effects of possible mediators on chronic kidney disease progression

The estimation of the effect of possible mediators on chronic kidney disease progression is shown in Figure 4(b) and Additional file 1: Table S4. In MVMR study, with adjustmention for GERD, genetics determine that each 1-unit increase in T2DM was associated with a 1.21-fold increased risk of CKD (OR = 1.21, 95% CI: 1.13–1.29), 1.18-fold kidney failure (OR = 1.18, 95% CI: 1.12–1.25), and 1.09-fold of dialysis-dependent kidney failure (OR = 1.09, 95% CI: 1.05–1.13)(all *p* < 0.05). With modification for GERD, genetics determine that each 1-unit increase in SBP was associated with a 1.01-fold increased risk of CKD (OR = 1.01, 95% CI: 1.00–1.02), 1.01-fold of kidney failure (OR = 1.01, 95% CI: 1.00–1.02), and 1.02-fold of dialysis-dependent kidney failure (OR = 1.02, 95% CI: 1.02–1.03) (all *p* < 0.05).

### Mediators in the associations of gastroesophageal reflux disease with chronic kidney disease progression

We employed two-step MR analyses to estimate the mediation pathway from GERD to chronic kidney disease progression *via* the potential mediators (Table 2). The T2DM mediated 43.24% of the total effect of GERD on CKD, followed by renal failure (32.47%) and dialysis-dependent kidney failure (14.33%). A similar mediation role was also found in the SBP.

**Table 2.** Mediation effects of GERD on CKD progression *via* mediators.

Mediators	Outcomes	Total effect β0 (95% CI)	Direct effect β1 (95% CI)	Mediation effect β2 (95% CI)	Mediated proportion % (95% CI)
SBP	CKD	0.17 (1.05, 1.33)	0.16 (1.04, 1.33)	0.01 (1.00, 1.02)	4.62 (−0.48, 9.71)
	Kidney failure	0.20 (1.09, 1.36)	0.19 (1.08, 1.35)	0.01 (1.00, 1.01)	3.85 (0.08,7.62)
	Dialysis-dependent kidney failure	0.23 (1.19, 1.34)	0.22 (1.17, 1.32)	0.01 (1.00, 1.02)	5.46 (1.72,9.21)
T2DM	CKD	0.17 (1.05, 1.33)	0.10 (0.97, 1.24)	0.07 (1.05, 1.11)	43.24 (26.36,60.11)
	Kidney failure	0.20 (1.09, 1.36)	0.13 (1.02, 1.28)	0.06 (1.04, 1.09)	32.47 (19.96, 44.98)
	Dialysis-dependent kidney failure	0.23 (1.19, 1.34)	0.20 (1.15, 1.30)	0.03 (1.02, 1.05)	14.33 (7.32, 21.34)

GERD: gastroesophageal reflux disease; CKD: chronic kidney disease; SBP: systolic blood pressure; T2DM: type 2 diabetes mellitus.

## Discussion

On the one hand, previous meta-analyses have suggested that metabolic syndrome is a risk factor for GERD [21]. On the other hand, Kidney Disease: Improving Global Outcomes (KDIGO) has also listed metabolic disorders as one of the key factors driving CKD in its CKD risk factors framework [22]. The results of this study are highly consistent with the conclusion of Wang et al. [3], and further provide strong evidence for the association between metabolic-related abnormalities and the risk of GERD-CKD.

These results support the hypothesis that GERD exerts a potential causal damage effect on CKD progression, whereby several changeable risk factors substantially mediate this effect, highlighting their critical role in the underlying mechanistic pathway. Targeted interventions on these modifiable factors thus hold the potential to alleviate the burden of CKD progression attributable to GERD dramatically. Identifying new therapeutic targets for CKD prevention holds significant clinical importance, offering innovative approaches to explore novel prevention and treatment strategies. This breakthrough addresses the gap in traditional nephropathy management regarding ‘cross-organ correlation’, while establishing a robust theoretical foundation for implementing multisystem integrated therapies. Furthermore, it enables early risk assessment and precision intervention targeting high-risk populations, providing concrete evidence to inform public health policymaking.

This present study explored the causal relationship between GERD and CKD progression, as well as demonstrated that GERD was causally associated with a 1.18-fold increased risk of CKD, 1.23-fold kidney failure, and 1.26-fold dialysis-dependent kidney failure. These discoveries find a potentially remarkable positive correlation between GERD and the risk of CKD progression, which includes CKD, kidney failure, and dialysis-dependent kidney failure. The causal relationships were not bidirectional because our results failed to find evidence that CKD progression had effects on GERD. In order to enhance the credibility of the conclusions, we employed a variety of sensitivity analyses. The consistency among the majority of WM, Weighted mode, MR-Egger, and IVW methods further verified the robustness of the research findings. Although some of the outcomes show relatively wide confidence intervals, the overall trend of the associations remained stable. Moreover, the application of the MR-PRESSO method helped to identify and eliminate potential outliers, thus enhancing the reliability of the conclusions. Combined with MR-Egger and MR-PRESSO, the influence of horizontal multinearity on causal effect estimation can be more comprehensively evaluated and the reliability of causal inference can be improved.

These results are consistent with some conventional observational studies, which have shown that higher GERD is associated with an increased risk of CKD progression. The research by Karahan et al. indicates that among patients with advanced CKD (3–5) and end-stage kidney disease (ESRD) requiring dialysis, the incidence of GERD is significantly increased [8]. At present, there is no mechanism to explain the association between GERD and chronic kidney disease. It is possible to work through the following mechanisms, which are commonly known as ‘acid reflux’. Gastric acid, whose main component is hydrochloric acid, usually causes esophageal irritation and reflux symptoms [23]. As a rule, esophageal epithelial cells can maintain a steady electrochemical gradient of potential difference, but HCI can affect the potential gradient of the esophageal mucosa [24]. Disturbance of potential differences can result in base imbalance. These base imbalances may subsequently lead to abnormal kidney function and, over time, potentially progress to CKD.

Currently, proton-pump inhibitors and H2-blockers are mainly used to treat GERD. Use of proton-pump inhibitors and H2-blockers in GERD patients: There is no evidence that these drugs may indirectly slow the progression of CKD progression, and this question remains to be verified in future studies.

Mediation analysis showed that T2DM was a potential key mediator linking GERD with CKD progression. IVW Analysis showed that GERD had a significant causal effect on T2DM. Sensitivity analysis showed no heterogeneity and horizontal pleiotropy. The mediation effects analysis showed that 43.24% of the total impact of GERD on CKD and 32.47% of the total effects of kidney failure were mediated by T2DM. In MVMR, part of the effect of GERD on dialysis-dependent kidney failure is mediated by the mediating variable T2DM (the mediating effect accounts for 14.33%). In conclusion, this study supports a causal pathway whereby GERD indirectly influences CKD progression through T2DM, a potential key mediating variable. A similar mediation role was also found in the SBP. However, measurement errors in mediating variables and the complexity of mediation pathways may introduce uncertainty. Moreover, mediation pathways often involve multiple steps and interactions, which can lead to uncertainties in causal effects. To assess the impact of mediating uncertainty on causal effect estimation, we conducted sensitivity analyses. These analyses effectively highlight mediating uncertainties in Mendelian randomization studies, thereby enhancing the reliability and scientific validity of research findings.

Previous conventional observational studies have shown that higher T2DM is the leading cause of the increase in CKD incidence [25]. In the present, there is no mechanism to explain the mechanism of action between them. A previous MR study found that genetic liability to T2DM was associated with an increased risk of GERD [6,20^,^]. Research has shown that diabetes is connected to an increased risk of impaired kidney function [26]. In GERD, the prevalence of vagus nerve dysfunction remains remarkably high [24]. The vagal nerve dysfunction may trigger pathways involved in blood sugar regulation [27,28^,^]. Diagnosis of hyperglycemia can lead to a series of pathophysiological disorders, including hypertension, altered tubuloglomerular feedback, renal hypoxia, and podocyte injury, which ultimately lead to progressive glomerulosclerosis and decreased glomerular filtration rate [29]. CKD, diabetes and hypertension are important components of cardiometabolic comorbidity, and they interact and aggravate each other through pathophysiological pathways. Accelerate disease progression.

T2DM, as an actionable mediator variable, may reduce the risk of renal impairment in patients with GERD by regulating blood glucose levels. For example, effective management of blood glucose levels in patients with GERD may reduce the risk associated with impaired kidney function [26]^.^ Given the significant impact of CKD on global public health, the CKD guidelines published by Kidney Disease: Improving Global Outcomes (KDIGO) have been an important reference for clinical practice and research. This finding further confirms that T2DM can mediate the effect of GERD on CKD, which has important implications for the assessment and management of CKD. However, this finding has not been fully reflected in current clinical guidelines. Our findings strongly recommend that future updates of the KDIGO CKD guidelines should consider and incorporate our findings more fully, considering both our findings and the need for clinical practice. This will help to improve the accuracy of clinicians’ assessment of CKD patients, optimize treatment strategies, and ultimately improve the clinical outcomes of patients. At the same time, it will also encourage more research to pay attention to this field and further promote the improvement of CKD diagnosis and treatment.

In a bidirectional Mendelian randomization research, Wei et al. demonstrated that GERD was linked with an increased risk of SBP [30]. The clinical symptoms of GERD often cause chest pain and discomfort, which may trigger neuro-reflexes, leading to increased sympathetic nerve activity and, consequently, hypertension. Hypertension and chronic kidney disease are closely linked. Hypertension is mainly due to renal damage caused by ischemia, and intensive blood pressure control can effectively reduce the risk of chronic kidney disease [30,31].

This research has several notable strengths. Compared with traditional observational studies, MR Shows substantially greater robustness against residual confounding and measurement error [^,^^32^]. Genetic variations are allocated randomly and fixed at conception and are not influenced by subsequent environmental factors [32]. This study employed univariate and multivariate MR Methods and utilized many SNPS identified in the literature and derived from recent high-power GWAS studies. MVMR is an innovative extension of MR Analysis that not only enables the unraveling of causal relationships but also facilitates the disambiguation of directionality in established associations, thereby validly removing the possibility of reverse causation [32].

Current studies have shown that there are intricate relationships among CKD, diabetes, and hypertension. The results of this study showed that interventions targeting GERD and related mediators (T2DM and SBP) could reduce the risk of developing CKD. Future research needs to further analyze the complex interaction mechanisms and common pathophysiological pathways among diabetes, hypertension, and chronic kidney disease, aiming to develop more precise and efficient multi-target intervention strategies. Gastroenterology experts particularly focus on gastrointestinal complications in patients with these chronic diseases, endocrinology experts focus on the comprehensive regulation of metabolic disorders, and primary care physicians play a key role in health management. This collaborative model can effectively delay or prevent the common progression and adverse outcomes of these chronic diseases, ultimately improving the quality of life and long-term prognosis of patients.

On the other hand, this study is not without certain limitations. First, there may be ancestry differences that are not fully controlled for, which may lead to inaccurate estimates of causal effects. Since the sample was derived from a group of European ancestry, analyses based on summary statistics from populations may be subject to sample selection bias, which could constrain the generalizations of the findings to other ancestry populations. This is of particular importance for vulnerable populations, such as Black and Hispanic patients, who exhibit marked phenotypic and prognostic disparities in hypertension and CKD [33]. Second, individual-level data were not available for our Mendelian randomization study, which limited the depth of our analysis. For example, we were unable to perform hierarchical analyses. Ideally, we would expect to divide the glomerular filtration rate or creatinine into five groups according to the stage of CKD to explore differences between these groups. However, we were not successful in conducting such analyses because of the absence of necessary individual-level data. Third, significant heterogeneity was found in certain studies related to directional pleiotropy in the analysis. The confounding effect of recessive pleiotropy is one of the well-known limitations of MR Analysis [34]. The differential functional effects of genetic variants on risk factors may be a reason for the observed heterogeneity; thus, the pathobiology of kidney disease is itself highly heterogeneous[34].

## Conclusion

The Mendelian randomization analysis has indicated a cause-and-effect connection between the GEDR and increased risk of CKD, kidney failure, and dialysis-dependent kidney failure, whereas the reverse causality hypothesis did not hold. Importantly, T2DM and SBP act as mediators of the impact of GERD on CKD progression. More experimental and clinical studies are warranted to verify and extend our findings. Our findings identify GERD as a novel genetic risk factor for CKD progression, meriting integration into future risk prediction models, guidelines, and preventive strategies.

## Supplementary Material

Table S1 Of Supplementary Material 1.docx

Table S4 Of Supplementary Material 1.docx

Supplementary specification of supplementary Material 2.docx

Table S3 Of Supplementary Material 1.docx

Supplementary Material 2.xlsx

Table S2 Of Supplementary Material 1.docx

## Data Availability

Access to the dataset upon which this study is based must be sought from UK Biobank and FinnGen datas. The code used in the study analyses is available upon request from the authors.
