# Supplementary material for: Genetic evidence linking gastroesophageal reflux disease to chronic kidney disease and kidney failure: a two-step Mendelian randomization study
Source: Ren Fail. 2025 Nov 3;47(1):2577842. doi: 10.1080/0886022X.2025.2577842 (PMC12584835; doi:10.1080/0886022X.2025.2577842)
Supplement: Table S1 Of Supplementary Material 1.docx [file IRNF_A_2577842_SM1652.docx]

**Table S1. Two-sample MR estimations showing the effect of GERD on CKD progression**

| **Outcomes** | **Methods** | **OR (95%CI)** | ***P*-value** | **Q statistic** | ***P*-heterogeneity** | **Egger intercept** | ***P*-intercept** |
| --- | --- | --- | --- | --- | --- | --- | --- |
| CKD | IVW | 1.18 (1.05-1.33) | 5.04E-03 | 74.86 | 0.450 |  |  |
|  | MR-Egger | 1.31 (0.65-2.63) | 0.451 | 74.78 | 0.420 | -0.003 | 0.773 |
|  | Weighted median | 1.13 (0.95-1.35) | 0.174 |  |  |  |  |
|  | Weighted mode | 1.10 (0.74-1.63) | 0.631 |  |  |  |  |
|  | MR-PRESSO | 1.18 (1.05-1.33) | 6.43E-03 |  |  |  |  |
| Kidney failure | IVW | 1.23 (1.11-1.36) | 3.95E-05 | 74.93 | 0.384 |  |  |
|  | MR-Egger | 2.04 (1.10-3.77) | 2.66E-02 | 72.24 | 0.437 | -0.016 | 0.108 |
|  | Weighted median | 1.22 (1.06-1.41) | 6.34E-03 |  |  |  |  |
|  | Weighted mode | 1.51 (1.03-2.22) | 3.98E-02 |  |  |  |  |
|  | MR-PRESSO | 1.23 (1.11-1.36) | 1.03E-04 |  |  |  |  |
| Dialysis-dependent kidney failure | IVW | 1.26 (1.19-1.34) | 3.06E-14 | 96.18 | 0.043 |  |  |
|  | MR-Egger | 1.13 (0.79-1.61) | 0.512 | 95.65 | 0.039 | 0.004 | 0.527 |
|  | Weighted median | 1.28 (1.18-1.39) | 1.44E-09 |  |  |  |  |
|  | Weighted mode | 1.44 (1.16-1.77) | 1.14E-03 |  |  |  |  |
|  | MR-PRESSO | 1.26 (1.19-1.34) | 7.51E-11 |  |  |  |  |

OR (95%CI) represents the risk for outcomes associated with each 1-SD higher GERD. OR, odds ratio; CI, confidence interval; CKD, chronic kidney disease; IVW, inverse variance weighted; MR-PRESSO, Mendelian randomization pleiotropy residual sum and outlier.
