# Supplementary material for: Genetic evidence linking gastroesophageal reflux disease to chronic kidney disease and kidney failure: a two-step Mendelian randomization study
Source: Ren Fail. 2025 Nov 3;47(1):2577842. doi: 10.1080/0886022X.2025.2577842 (PMC12584835; doi:10.1080/0886022X.2025.2577842)
Supplement: Table S4 Of Supplementary Material 1.docx [file IRNF_A_2577842_SM1651.docx]

**Table S4. The estimates for causal effect of mediators on CKD progression**

| **Exposure** | **Outcome** | **Methods** | **OR (95%CI)** | ***P*-value** | **Q statistic** | ***P*-heterogeneity** | **Egger intercept** | ***P*-intercept** |
| --- | --- | --- | --- | --- | --- | --- | --- | --- |
| SBP | CKD | IVW | 1.01 (1.00, 1.02) | 0.023 | 286.54 | 2.42E-06 | 4.05E-04 | 0.854 |
|  | Kidney failure | IVW | 1.01 (1.00, 1.02) | 0.006 | 278.27 | 1.09E-05 | -7.59E-04 | 0.666 |
|  | Dialysis-dependent kidney failure | IVW | 1.02 (1.02, 1.03) | 2.39E-15 | 313.04 | 1.22E-08 | -2.47E-03 | 0.016 |
| T2DM | CKD | IVW | 1.21 (1.13, 1.29) | 7.75E-09 | 119.70 | 0.315 | -4.09E-04 | 0.878 |
|  | Kidney failure | IVW | 1.18 (1.12, 1.25) | 4.33E-09 | 133.73 | 0.089 | -9.15E-04 | 0.688 |
|  | Dialysis-dependent kidney failure | IVW | 1.09 (1.05, 1.13) | 1.24E-05 | 216.25 | 1.79E-08 | 2.28E-04 | 0.887 |
| BMI | CKD | IVW | 1.58 (1.31, 1.92) | 2.50E-06 | 586.78 | 1.17E-04 | 0.001 | 0.767 |
|  | Kidney failure | IVW | 0.99 (0.89, 1.10) | 0.842 | 830.43 | 7.03E-23 | 1.27E-04 | 0.926 |
|  | Dialysis-dependent kidney failure | IVW | 1.47 (1.26, 1.72) | 1.01E-06 | 579.06 | 2.69E-04 | -0.001 | 0.791 |
| Whole body fat mass | CKD | IVW | 1.23 (1.02, 1.49) | 0.034 | 194.86 | 0.683 | -0.002 | 0.276 |
|  | Kidney failure | IVW | 0.93 (0.82, 1.06) | 0.268 | 448.07 | 3.48E-20 | -0.001 | 0.722 |
|  | Dialysis-dependent kidney failure | IVW | 1.23 (1.06, 1.44) | 0.008 | 204.46 | 0.498 | -0.002 | 0.282 |
| Body fat percentage | CKD | IVW | 1.11 (0.80, 1.53) | 0.524 | 235.61 | 0.006 | -0.002 | 0.543 |
|  | Kidney failure | IVW | 0.86 (0.71, 1.05) | 0.133 | 410.67 | 2.39E-19 | -0.001 | 0.793 |
|  | Dialysis-dependent kidney failure | IVW | 1.19 (0.92, 1.55) | 0.185 | 234.69 | 0.007 | -0.002 | 0.399 |
| Trunk fat mass | CKD | IVW | 0.82 (0.68, 0.99) | 0.041 | 192.46 | 0.010 | -0.004 | 0.252 |
|  | Kidney failure | IVW | 1.10 (0.98, 1.24) | 0.102 | 358.15 | 2.95E-19 | -0.001 | 0.444 |
|  | Dialysis-dependent kidney failure | IVW | 0.83 (0.71, 0.97) | 0.017 | 194.46 | 0.007 | -0.003 | 0.279 |
| Hypertension | CKD | IVW | 3.76 (0.92, 15.41) | 0.066 | 118.67 | 0.016 | 0.009 | 0.076 |
|  | Kidney failure | IVW | 9.31 (4.88, 17.79) | 1.38E-11 | 122.96 | 0.008 | 0.005 | 0.023 |
|  | Dialysis-dependent kidney failure | IVW | 4.36 (1.27, 14.92) | 0.019 | 137.18 | 0.001 | 0.005 | 0.261 |

OR (95%CI) represents the risk for outcomes associated with each 1-SD mediator. OR, odds ratio; CI, confidence interval; SBP, systolic blood pressure; IVW, inverse variance weighted; T2DM, type 2 diabetes mellitus; BMI, body mass index.
