# Supplementary material for: Genetic evidence linking gastroesophageal reflux disease to chronic kidney disease and kidney failure: a two-step Mendelian randomization study
Source: Ren Fail. 2025 Nov 3;47(1):2577842. doi: 10.1080/0886022X.2025.2577842 (PMC12584835; doi:10.1080/0886022X.2025.2577842)
Supplement: Supplementary specification of supplementary Material 2.docx [file IRNF_A_2577842_SM1650.docx]

Supplementary specification of supplementary Material 2

In the outcome column of table in the supplementary material 2, "renal failure" has been replaced by "kidney failure" and "dialysis" by "dialysis-dependent kidney failure" in the text.
