# Supplementary material for: Genetic evidence linking gastroesophageal reflux disease to chronic kidney disease and kidney failure: a two-step Mendelian randomization study
Source: Ren Fail. 2025 Nov 3;47(1):2577842. doi: 10.1080/0886022X.2025.2577842 (PMC12584835; doi:10.1080/0886022X.2025.2577842)
Supplement: Table S3 Of Supplementary Material 1.docx [file IRNF_A_2577842_SM1649.docx]

**Table S3. The estimates for causal effect of GERD on mediators**

| **Outcomes** | **Methods** | **β (95%CI)** | ***P*-value** | **Q statistic** | ***P*-heterogeneity** | **Egger intercept** | ***P*-intercept** |
| --- | --- | --- | --- | --- | --- | --- | --- |
| BMI | IVW | 1.14 (1.11, 1.17) | 2.14E-20 | 40.39 | 0.036 | 0.007 | 0.073 |
| Whole body fat mass | IVW | 1.16 (1.12, 1.20) | 1.14E-19 | 71.48 | 2.67E-04 | 4.38E-03 | 0.234 |
| Body fat percentage | IVW | 1.13 (1.11, 1.15) | 1.77E-32 | 39.19 | 0.178 | 2.87E-03 | 0.296 |
| Trunk fat mass | IVW | 0.87 (0.83, 0.92) | 2.03E-08 | 121.44 | 1.18E-10 | 3.60E-04 | 0.949 |
| Hypertension | IVW | 1.04 (1.04, 1.05) | 3.92E-30 | 123.15 | 1.81E-05 | 1.67E-03 | 0.020 |
| SBP | IVW | 1.90 (1.26, 2.86) | 2.01E-03 | 180.04 | 9.65E-13 | 0.019 | 0.656 |
| T2DM | IVW | 1.47 (1.36, 1.58) | 5.40E-25 | 115.12 | 3.15E-04 | -6.44E-03 | 0.418 |
| Body fat percentage | IVW | 0.89 (0.86, 0.92) | 3.32E-10 | 98.46 | 3.47E-08 | -1.10E-03 | 0.828 |
| DBP | IVW | 1.25 (0.97, 1.59) | 0.080 | 199.69 | 1.29E-15 | 0.006 | 0.808 |
| Total cholesterol | IVW | 1.02 (0.96, 1.08) | 0.475 | 142.14 | 7.29E-08 | 8.42E-03 | 0.108 |

OR (95%CI) represents the risk for mediators associated with each 1-SD higher GERD. OR, odds ratio; CI, confidence interval; BMI, body mass index; IVW, inverse variance weighted; SBP, systolic blood pressure; T2DM, type 2 diabetes mellitus; DBP, Diastolic blood pressure.
