# Supplementary material for: Genetic evidence linking gastroesophageal reflux disease to chronic kidney disease and kidney failure: a two-step Mendelian randomization study
Source: Ren Fail. 2025 Nov 3;47(1):2577842. doi: 10.1080/0886022X.2025.2577842 (PMC12584835; doi:10.1080/0886022X.2025.2577842)
Supplement: Table S2 Of Supplementary Material 1.docx [file IRNF_A_2577842_SM1647.docx]

**Table S2. Bidirectional MR estimations showing the effect of CKD progression on GERD**

| **Exposure** | **Methods** | **β (95%CI)** | ***P*-value** | **Q statistic** | ***P*-heterogeneity** | **Egger intercept** | ***P*-intercept** |
| --- | --- | --- | --- | --- | --- | --- | --- |
| CKD | IVW | 0.98 (0.90-1.06) | 0.564 | 9.92 | 0.078 |  |  |
|  | MR-Egger | 0.86 (0.62-1.19) | 0.407 | 8.51 | 0.075 | 0.014 | 0.461 |
|  | Weighted median | 0.94 (0.87-1.02) | 0.134 |  |  |  |  |
|  | Weighted mode | 0.92 (0.80-1.05) | 0.280 |  |  |  |  |
|  | MR-PRESSO | 0.98 (0.90-1.06) | 0.589 |  |  |  |  |
| Kidney failure | IVW | 1.01(0.91-1.11) | 0.917 | 7.01 | 0.135 |  |  |
|  | MR-Egger | 0.90 (0.51-1.60) | 0.747 | 6.69 | 0.082 | 0.008 | 0.730 |
|  | Weighted median | 1.07 (0.97-1.19) | 0.175 |  |  |  |  |
|  | Weighted mode | 1.08 (0.95-1.22) | 0.301 |  |  |  |  |
|  | MR-PRESSO | 1.01 (0.91-1.11) | 0.922 |  |  |  |  |
| Dialysis-dependent kidney failure | IVW | 0.98 (0.93-1.03) | 0.364 | 58.25 | 1.12E-04 |  |  |
|  | MR-Egger | 0.93 (0.85-1.01) | 0.092 | 53.09 | 3.56E-04 | 0.005 | 0.148 |
|  | Weighted median | 0.97 (0.93-1.02) | 0.298 |  |  |  |  |
|  | Weighted mode | 0.96 (0.92-1.01) | 0.109 |  |  |  |  |
|  | MR-PRESSO | 0.98 (0.93-1.03） | 0.373 |  |  |  |  |

OR (95%CI) represents the risk for GERD associated with each 1-SD higher CKD progression. OR, odds ratio; CI, confidence interval; CKD, chronic kidney disease; IVW, inverse variance weighted; MR-PRESSO, Mendelian randomization pleiotropy residual sum and outlier.
